# Characterization and phylogenetic analysis of the complete chloroplast genome of *Camellia chrysanthoides* (Theaceae)

**DOI:** 10.1080/23802359.2021.1981788

**Published:** 2021-09-29

**Authors:** Yupeng Wang, Xinlei Li, Hengfu Yin, Jiyuan Li, Zhengqi Fan, Weixin Liu

**Affiliations:** a College of Information Science and Technology, Nanjing Forestry University, Nanjing, China; b State Key Laboratory of Tree Genetics and Breeding, Research Institute of Subtropical, Forestry, Chinese Academy of Forestry, Hangzhou, China

**Keywords:** *Camellia chrysanthoides*, chloroplast genome, phylogenetic analysis

## Abstract

*Camellia chrysanthoides* H.T.Chang 1979 is an economic species for its high ornamental and medicinal values. In the present work, the complete chloroplast (cp) genome sequence of *C. chrysanthoides* was assembled and characterized using Illumina paired-end sequencing data. The total cp genome of *C. chrysanthoides* is 157,439 bp in size, consisting of a small single copy (SSC) and a large single copy (LSC) separated by a pair of inverted repeats (IRs) with 18,265 bp, 88,162 bp, and 25,506 bp, respectively. Further annotation revealed the cp genome encoded 124 genes, including 82 protein-coding genes, 34 tRNA genes, and eight rRNA genes, and the overall GC content of the cp genome is 37.31%. Phylogenetic analysis based on 80 protein-coding genes shows that *C. chrysanthoides*is closely related to *C. azalea* in the genus *Camellia*.

*Camellia chrysanthoides* H.T.Chang 1979 has high ornamental value and breeding value for the flowers are golden yellow and the young leaves are light mauve (Zhang 1998). *Camellia chrysanthoides* is a rare variety with golden color flowers in Theaceae, which can be used to cultivate new varieties. *C. chrysanthoides* is a Chinese herb for the flowers and leaves of are rich in natural nutrients such as tea polyphenols and brass that are beneficial to the human body (Chen et al. 2019). Here, we report the complete cp genome of *C. chrysanthoides* (Genbank accession: MZ618349), which will provide favorable tools for researching the phylogenetic and origination of different *Camellia* plants.

The samples of *Camellia chrysanthoides* were collected from Jinhua International Camellia Species Park (Coordinates: 29°7′10.1208″N, 119°35′52.1088″E), Zhejiang Province, China. The voucher specimen and DNA sample (BY1001, BY1001_DNA) were deposited at State Key Laboratory of Tree Genetics and Breeding, Research Institute of Subtropical Forestry, Chinese Academy of Forestry (http://risfcaf.caf.ac.cn/; Xinlei Li, lixinlei2020@163.com). Total genomic DNA was extracted from fresh and healthy leaves by MiniBEST plant Genomic DNA Extraction Kit (Takara, Dalian, China). After library preparation, the DNA library was sequenced on the Illumina HiSeq 4000 sequencing system (Illumina, San Diego, California, USA), and we obtained 28,770,636 raw reads. The low-quality reads were filtered by Trimmomatic (Bolger et al. 2014). The remaining 27,186,810 clean reads were used to assemble the cp chloroplast genome by NOVOplasty v4.3.1 (Dierckxsens et al. 2017) with the complete cp genome of *Camellia nitidissima* (Genbank accession: MH382827.1) as the reference. The assembled cp genome was annotated by PGA (Qu et al. 2019) and Geneious v11.0.3 (Kearse et al. 2012), and manually correct the annotations based on Blast search result.

The complete cp genome of *Camellia chrysanthoides* is a typical quadripartite structure (Wang et al. 2018) of 157,439 bp in length, consisting of an SSC region of 18,265 bp, an LSC region of 88,162 bp, and a pair of IRs region of 25,506 bp. The overall GC context of cp DNA is 37.31%, and the corresponding GC values of the SSC, LSC, and IRs are 30.60%, 35.42%, and 42.97% respectively. The cp genome contains 124 predicted functional genes, including 82 protein-coding genes, 34 tRNA genes, and eight rRNA genes. In total, there are 15 intron-containing genes, including eight protein-coding genes and seven tRNA genes. Fourteen genes comprise one intron and only one gene (*clpP*) comprise two introns.

To investigate the phylogenetic status of the cp genome of *Camellia chrysanthoides*, a Neighbor-joining (NJ) tree based on 80 protein-coding genes was reconstructed by MEGA v7.0.147 (Kumar et al. 2016). *Camellia chrysanthoides* and other 14 published complete cp genome from the genus *Camellia* were included as in-group taxa, and *Stewartia micrantha* (NC_041471.1) and *Stewartia obovata* (NC_041472.1) were used as the outgroup. The NJ tree was based on the Jones-Taylor-Thornton (JTT) model and 1000 bootstrap replicates. As illustrated in Figure 1, the cp genome of *C. chrysanthoides* is clustered closely to *Camellia azalea* (NC_035574.1).

**Figure 1.**
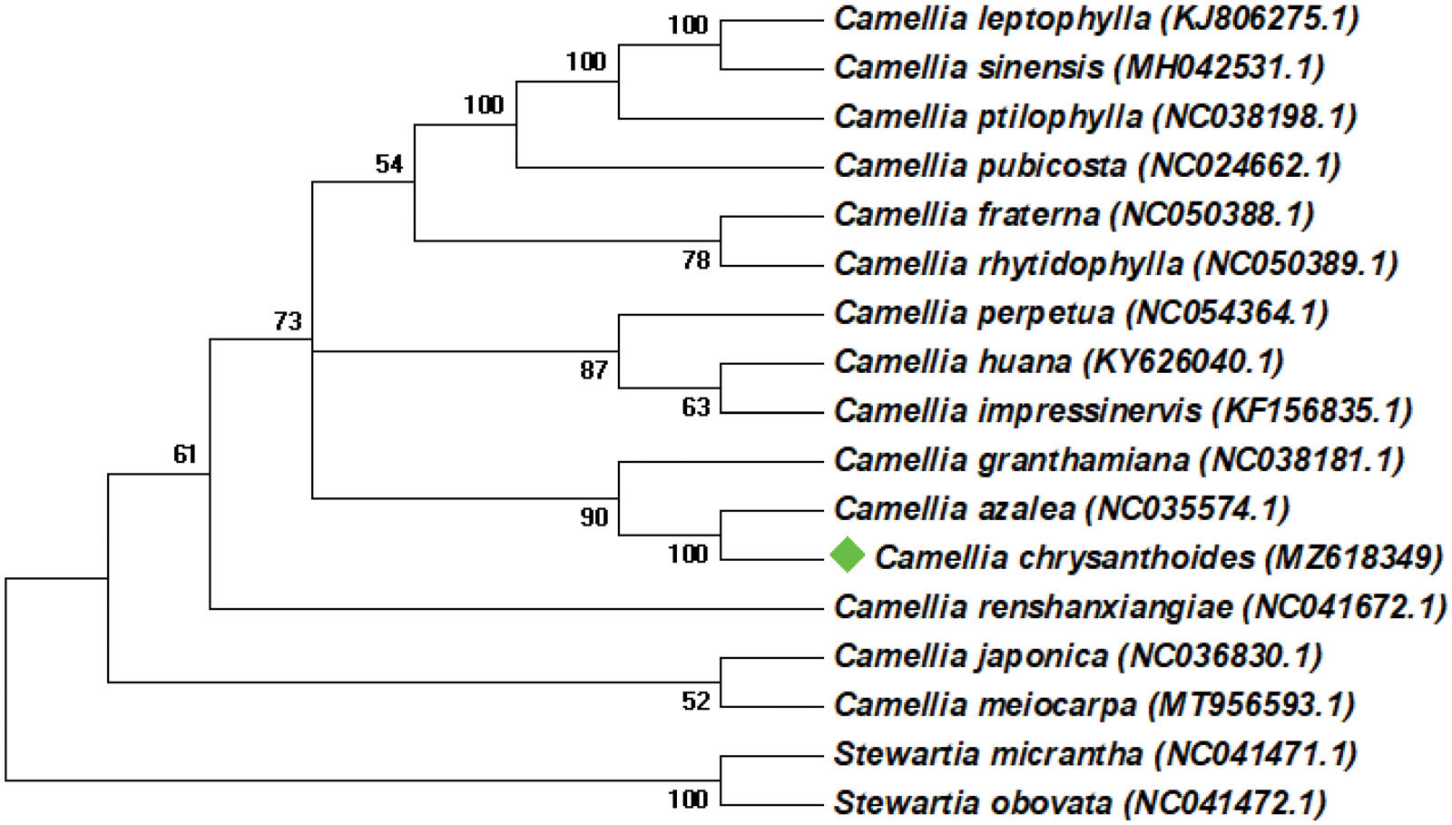
The Neighbor-joining tree based on 80 protein-coding genes were conducted in MEGA v7.0.147. The bootstrap support values > 50% from 1000 replicates are listed for each node.

## Data Availability

The genome sequence data that support the findings of this study are openly available in GenBank of NCBI at [https://www.ncbi.nlm.nih.gov] under the accession No. MZ618349. The associated BioProject, SRA, and Bio-Sample numbers are PRJNA748453, SRR15204590, and SAMN20335808 respectively.
